# Tracing the structural evolution of quasi-freestanding germanene on Ag(111)

**DOI:** 10.1038/s41598-022-10943-0

**Published:** 2022-05-09

**Authors:** Lukas Kesper, Julian A. Hochhaus, Marie Schmitz, Malte G. H. Schulte, Ulf Berges, Carsten Westphal

**Affiliations:** 1 Department of Physics, TU Dortmund University, 44227 Dortmund, Germany Otto-Hahn-Str. 4a,; 2 DELTA, Center for Synchrotron Radiation, TU Dortmund University, 44227 Dortmund, Germany Maria-Goeppert-Mayer-Str. 2,

**Keywords:** Materials science, Condensed-matter physics, Nanoscale materials, Structural materials

## Abstract

In the last decade, research on 2D materials has expanded massively due to the popularity of graphene. Although the chemical engineering of two-dimensional elemental materials as well as heterostructures has been extensively pursued, the fundamental understanding of the synthesis of 2D materials is not yet complete. Structural parameters, such as buckling or the interface structure of a 2D material to the substrate directly affect its electronic characteristics. In order to proceed the understanding of the element-specific growth and the associated ability of tuning material properties of two-dimensional materials, we performed a study on the structural evolution of the promising 2D material germanene on Ag(111). This study provides a survey of germanium formations at different layer thicknesses right up to the arising of quasi-freestanding germanene. Using powerful surface analysis tools like low-energy electron diffraction, x-ray photoelectron spectroscopy, and x-ray photoelectron diffraction with synchrotron radiation, we will reveal the internal and interfacial structure of all discovered germanium phases. Moreover, we will present models of the atomic and chemical structure of a $\hbox {Ag}_2\hbox {Ge}$ surface alloy and the quasi-freestanding germanene with special focus on the structural parameters and electronic interaction at the interface.

## Introduction

Germanene, the two-dimensional counterpart of germanium, has attracted worldwide attention in the course of the first synthesis and analysis of graphene. It gave the go-ahead to a new generation of chemical engineering of 2D materials, which became famous for their outstanding electronic properties, as for example their linear Dirac-like dispersions and extraordinary high charge carrier mobilities^1,2^. Turning to two-dimensional, semiconducting materials with increasing atomic number, like silicene, germanene, stanene, etc., it additionally opens the opportunity to take advantage of topological insulators and spin effects, paving the way for high-speed nanoelectronics^3,4^. Concerning next-generation computing technologies, it is inevitable to consider new materials for the fabrication of field-effect transistors (FET) with feature sizes below ${5}\,\hbox {nm}$^5^. Apart from the multitude of graphene-based applications^6^, even astonishingly performing silicene- and germanene-based transistors have recently been realized^7,8^. Facing the challenges of bringing promising 2D materials to fabrication, it is necessary to improve the understanding of the synthesis and structural formation of these materials.

First-principle calculations determined a stable, two-dimensional phase of germanium^9^, arranged in a low-buckled honeycomb structure with promising electronic properties^10^. The charge carriers in germanene behave like massless Dirac fermions, whose mobility may be a factor 2 larger than for the metal-like graphene^10,11^. On the other hand, the strong spin-orbit coupling of germanene opens a band gap that can additionally be tuned by an external electric field^10,12^. One way to control the electronic properties of germanene for applications is to take hold on a structural key parameter, the buckling. This corrugation of the honeycomb lattice was calculated to be $\delta ={0.69}$ Å for freestanding germanene^13^, since its size is directly correlated to the share of $sp^2$- and $sp^3$-hybridizied bonds^14^. While a high buckling effects a large band gap in germanenes band structure^15^, also low-buckled germanene is of great interest, since the band gap is still larger than silicene’s and the quantum spin Hall effect (QSHE) can be realized^16^. Moreover, the magnitude of the buckling in germanene strongly depends on the on-growing carrier substrate^17^. Ag(111) turned out to be a promising candidate for synthesizing freestanding germanene by considering the encouraging predictions of Dirac cones in germanene on silver^18^, as well as moderate interaction and poor charge transfer at the interface compared to other substrates^19–21^. But even germanium alloy phases on Ag(111) feature Dirac signatures in their electronic structure^22^.

The first experimental study on thin films of epitaxially grown germanium on Ag(111) mainly reported a surface alloy at a coverage of $1/3\,\hbox {ML}$, arranged in a $(\sqrt{3}\times \sqrt{3})\mathrm {R}30^\circ$ reconstruction^23,24^. However, this $\mathrm {Ag}_2\mathrm {Ge}$ alloy phase at a layer thickness of $1/3\,\hbox {ML}$ germanium is still controversially discussed to form reconstructions of a $(\sqrt{3}\times \sqrt{3})\mathrm {R}30^\circ$^25–28^, a $(9\sqrt{3}\times 9\sqrt{3})\mathrm {R}30^\circ$^29^, a $(\frac{19}{20}\sqrt{3}\times \frac{19}{20}\sqrt{3})\mathrm {R}30^\circ$ with a Moiré pattern of $(19\sqrt{3}\times 19\sqrt{3})\mathrm {R}30^\circ$^30^, or a rectangular $\mathrm {Rec}\{c(31\times \sqrt{3})\}$^31^. It was also considered as a striped phase (SP) due to tensile strains of the uppermost layer^32^ with a long-range order of $(6\sqrt{3}\times \sqrt{3})\mathrm {R}30^\circ$^26^. Then, after passing a mixed phase, the germanene arranges at a coverage of ${1.06}\,\hbox {ML}$^32^, whose superstructure might be described as a $(7\times 7)$^23^, a $\mathrm {Rec}\{c(\sqrt{3}\times 7)\}$^29,33^, or a $(1.35\times 1.35)\mathrm {R}30^\circ$^34,35^ with a long-range order of $(7\sqrt{7}\times 7\sqrt{7})\mathrm {R}19.1^\circ$^34,36^. The large number of recently published controversial studies and results on what phases are formed and what they are called do not proceed the understanding of the structural formation process of germanene on Ag(111). Above all, the critical discussion of controversial structural models proposed, as also known from other materials, is highly topical and contemporary for the progress in the field of 2D materials^37–40^.

To add clarity on this particular issue, we present our experimental study on the structural evolution towards the formation of quasi-freestanding germanene on Ag(111). We used low-energy electron diffraction (LEED) to sketch the evolution of different phases of germanium formations with increasing layer thicknesses. To make a clear distinction between all phases, we resolved both the internal and the interfacial electronic and chemical structures of the germanium films by performing high-resolution photoelectron spectroscopy (XPS) of the Ge 3d and Ag 3d core-level with synchrotron excitation. In combination with measurements of photoelectron diffraction (XPD), we will propose structural models for a surface alloy phase, as well as the quasi-freestanding phase of germanene.

## Results and discussion

**Figure 1:**
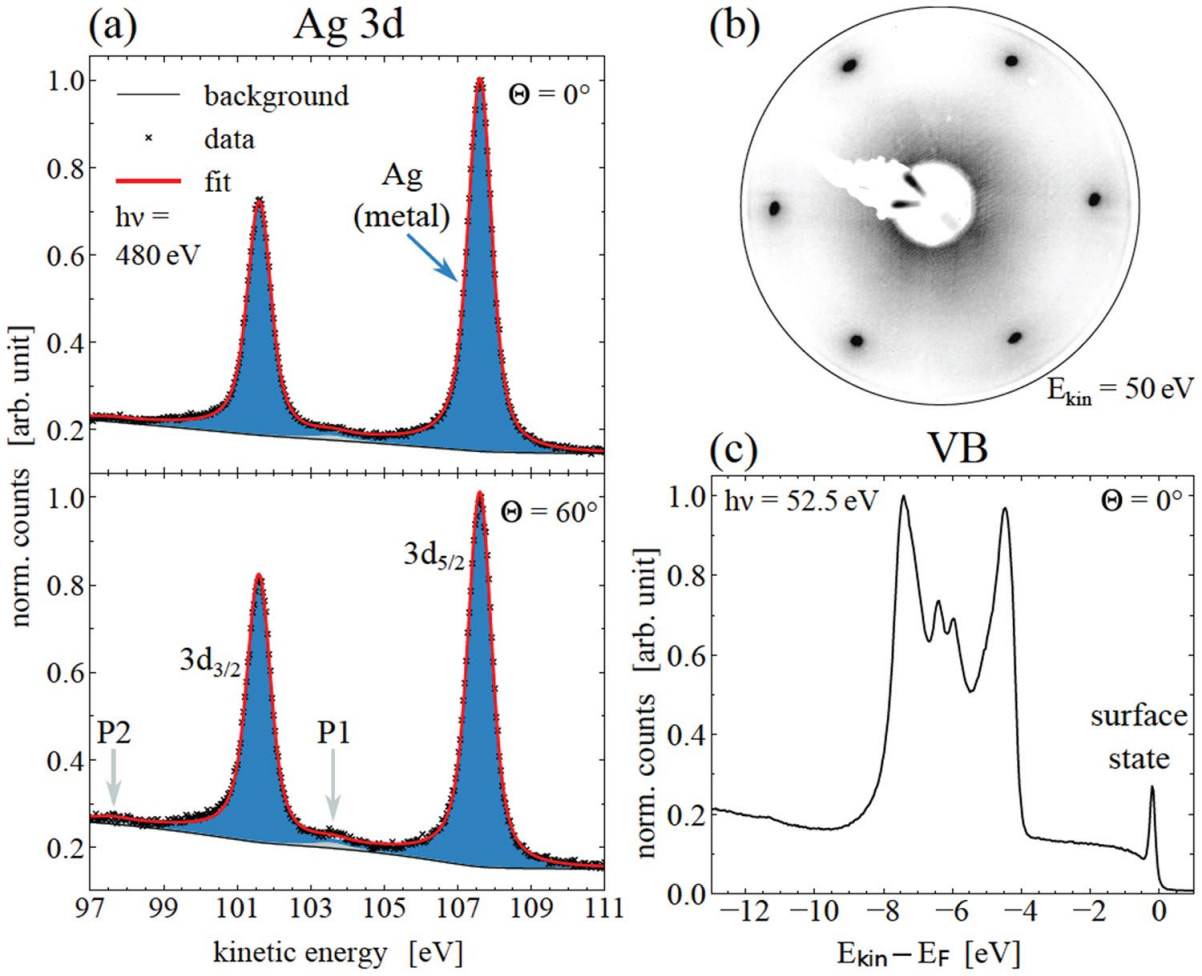
Reconstructed clean Ag(111) substrate. (**a**) High-resolution XPS spectra of the Ag 3d signal of the Ag(111) sample, recorded at a photon energy of $\mathrm {h}\nu = {480}\,\hbox {eV}$ and under emission angles of $\Theta ={0}^{\circ }$ (top) and $\Theta ={60}^{\circ }$ (bottom). (**b**) The corresponding LEED pattern was obtained with $E_\mathrm {kin} = {50}\,\hbox {eV}$, and (**c**) the high-resolution VB spectra at $\mathrm {h}\nu = {52.5}\,\hbox {eV}$ under normal emission.

The sufficient preparation of the Ag(111)-surface was checked for its long-range order by LEED and for its chemical purity by XPS survey and high-resolution spectra. After several preparation cycles the following results were obtained, as shown in Fig. 1. The LEED pattern revealed sharp reflection spots of the $(1\times 1)$ reconstruction, which is displayed in Fig. 1b.

The well-prepared surface can also be evaluated by indicators like a Shockley surface state in the high-resolution spectra of the valence band (VB) in subfigure (c)^24,41^, as well as surface plasmons, which are identified in the angle-resolved XPS spectra of the Ag 3d core-level^42^. Table 1 shows the fit parameters of Fig. 1a. Due to the electronic structure of noble metals, the peak shape of the metallic Ag 3d core-level is slightly asymmetric with an asymmetry parameter of $\alpha ={0.02}$^43^. Moreover, no signal or chemical bond of contaminating residues was noted.

**Table 1:** Fit parameters within the XPS analysis of asymmetric components, applied to the Ag 3d core-level signal in Fig. 1a.

Figure 1	$\Theta$ $(^{\circ })$	Comp.	$E_\mathrm {kin}$ $(\hbox {eV})$	$E_\mathrm {SOC}$ $(\hbox {eV})$	$\mathrm {FWHM}$ $(\hbox {eV})$	$\alpha$	$A_\mathrm {rel}$ $(\%)$
(a)	0	Ag	107.61	6.00	0.78	0.02	98.7
		P1	103.56		1.13	0.00	0.9
		P2	97.55		1.12	0.00	0.4
	60	Ag	107.61	6.00	0.78	0.02	98.1
		P1	103.56		1.13	0.00	1.1
		P2	97.55		1.12	0.00	0.8

All parameters refer to the Ag 3d$_{5/2}$ peak.

### LEED

**Figure 2:**
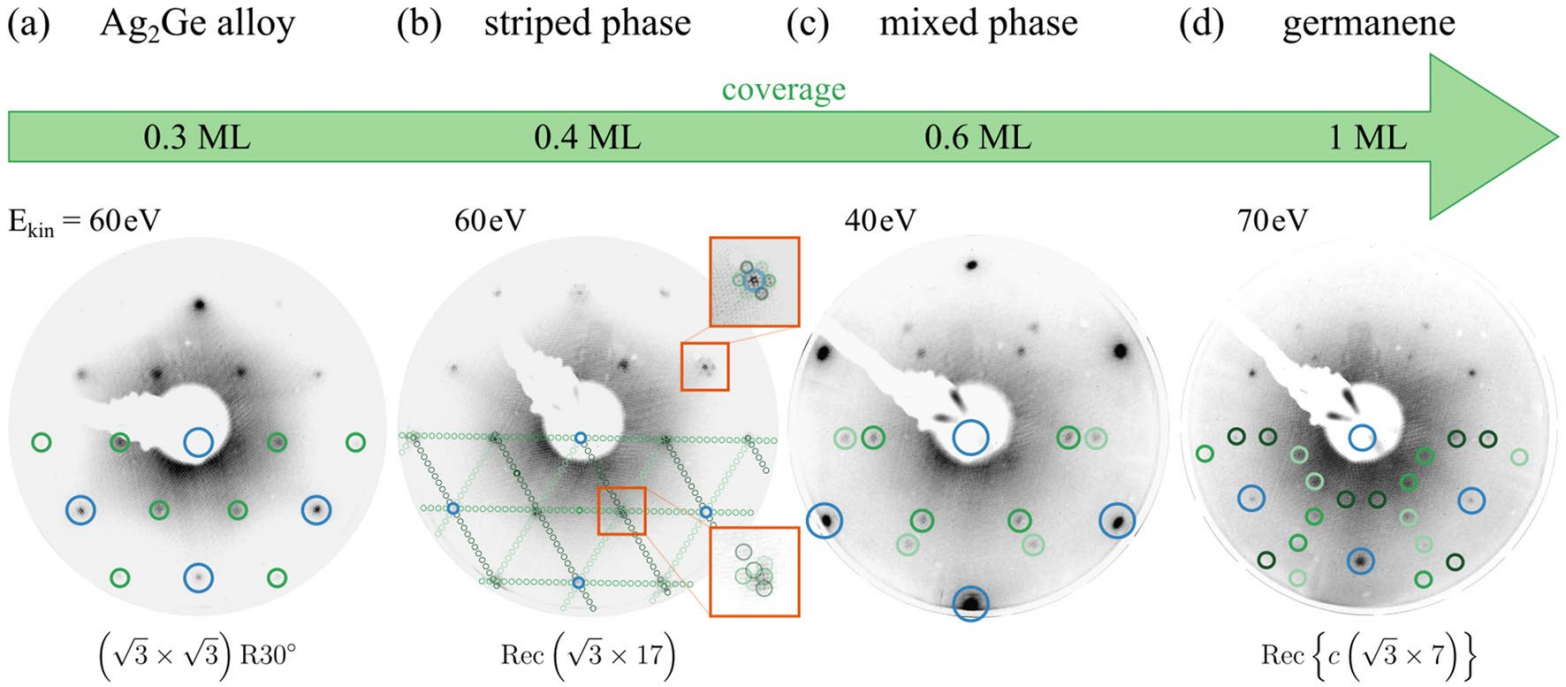
Overview of all phases with respective LEED patterns at different coverages. The marked reflection spots were obtained from *LEEDpat* simulations, while the blue circles correspond to the Ag lattice and circles in shades of green indicate the Ge spots of different domains.

In the following, the clean Ag(111) surface was covered by thin films of germanium. The layer thickness was stepwise increased until a modification of the LEED pattern was observed. By this procedure, the different Ge phases were found, as presented in Fig. 2. To determine the suitable description of the formed Ge superstructure, the periodicity was simulated using *LEEDpat*^44^. The locations of the diffraction spots obtained from the simulation were added by colored circles to the lower half of each measured LEED pattern.

For low coverages of $<1/3\,\hbox {ML}$ germanium on the substrate’s surface no formation of periodic superstructures could be observed in the respective LEED pattern. However, a clear $(\sqrt{3}\times \sqrt{3})\mathrm {R}{30}^{\circ }$ reconstruction was recognized at a coverage of $1/3\,\hbox {ML}$, as displayed in Fig. 2a. According to similar reports of this superstructure, mainly obtained from LEED and STM measurements, the formation can be attributed to a $\hbox {Ag}_2\hbox {Ge}$ surface alloy^22,24–28^. Its particular structure will be discussed in “XPD”. Fig. 2b shows the LEED pattern after increasing the Ge coverage slightly to approximately ${0.4}\,\hbox {ML}$. Some satellite features around the substrate spots and the spots of the $(\sqrt{3}\times \sqrt{3})$-reconstruction were observed, which are attributed to a tensile strain along the zigzag and the armchair direction of the Ge-superstructure, as reported by Lin et al.^32^. This strain leads to a modulation in the electron density which was observed as stripes in several STM studies^26,28,32,35,45^. Many descriptions of this superstructure for the striped phase have been published^26,29,31^. Here, a superstructure of $\mathrm {Rec}(\sqrt{3}\times 17)$ with three domains fully satisfies the LEED pattern, as visualized in Fig. 2b. Following the idea of the $(\sqrt{3}\times 22)$-herringbone reconstruction of Au(1 1 1), this $\mathrm {Rec}(\sqrt{3}\times 17)$-superstructure with matrix notation $$\left(\begin{array}{*{20}{c}} 17 &{} 0 \\ -1 &{} 2\end{array} \right)$$ was obtained. The periodicity found is caused by a compression of the uppermost $\hbox {Ag}_2\hbox {Ge}$ layer, which fits exactly into mold of a structural model of the striped phases proposed by Zhang et al.^31^. The complex but significant distinction between the $\hbox {Ag}_2\hbox {Ge}$ alloy phase and the striped phase will be discussed further in “XPS” by their electronic structure. The transition from the striped phase to germanene is effected by means of coexisting alloy- and germanene-phases^32^. This mixed phase shows its most distinct LEED pattern, presented in Fig. 2c, at a layer thickness of ${0.6}\,\hbox {ML}$, which is a superposition of the $(\sqrt{3}\times \sqrt{3})$ and the germanene’s superstructures. However, the germanene phase, also called quasi-freestanding germanene^32,35^, forms at a coverage of approximately ${1}\,\hbox {ML}$. The corresponding LEED pattern is presented in Fig. 2d. Even if some patterns at certain kinetic energies agree with a proposed short-range order of a $(1.35\times 1.35)\mathrm {R}{30}^{\circ }$^34^, not all spots are included. The superstructure of the germanene is superimposed with a Moiré pattern^29,32^, resulting in a $\mathrm {Rec}\{c(\sqrt{3}\times 7)\}$ structure^29,33^ with three domains. The matrix notation is $$\left( \begin{array}{*{10}{c}} 1 &{} 1 \\ -3 &{} 4 \end{array}\right)$$ , while some spots are strongly attenuated by multiple electron scattering processes^33^. Based on our finding about the long-range structure of the germanium phases, the investigation of the structural evolution will proceed with a detailed analysis of the chemical structure.

### XPS

**Figure 3:**
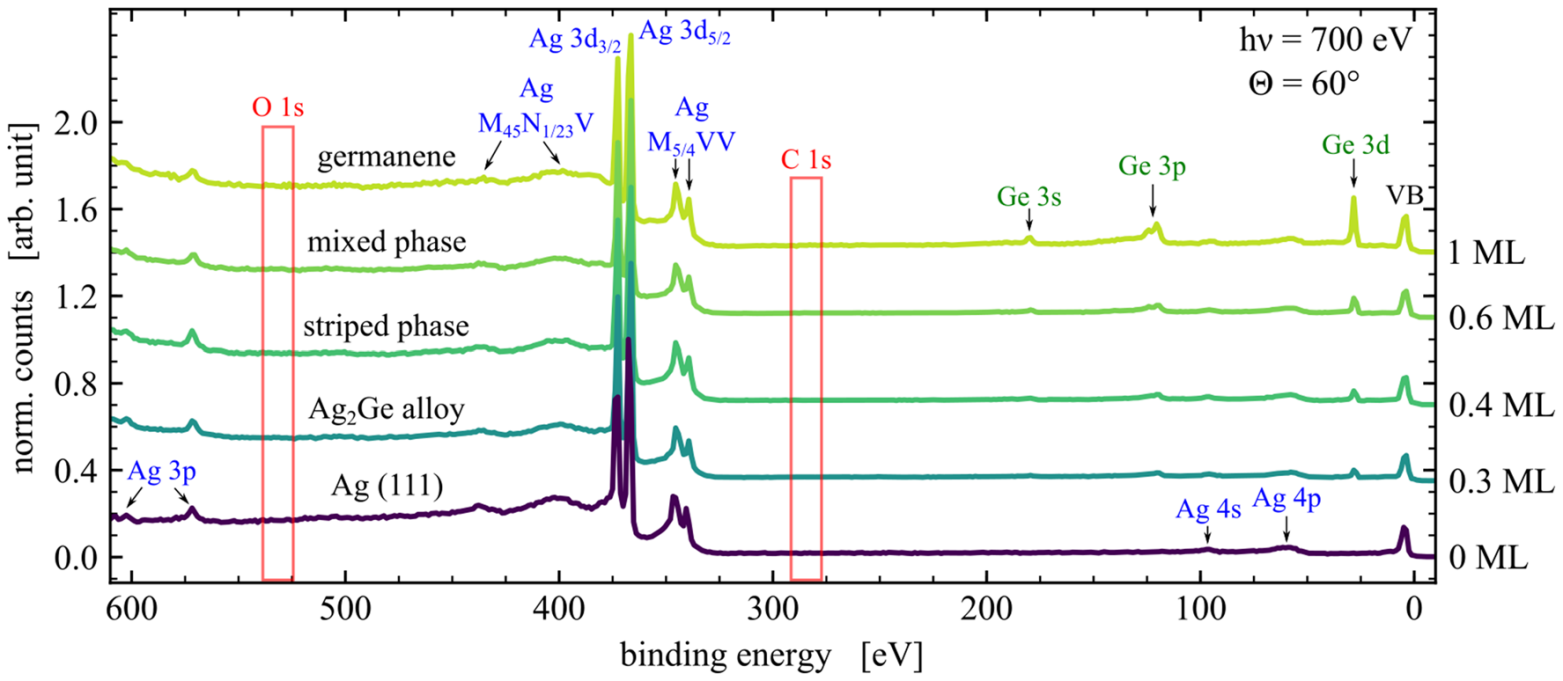
XPS survey spectra. The data were obtained for the clean Ag(111) sample and all Ge-phases at different coverages, taken at $\mathrm {h}\nu ={700}\hbox { eV}$ and $\Theta ={60}^{\circ }$. The binding energy of the spectra was referenced to the Fermi edge.

In order to get an overview of the chemical composition of all discovered phases, XPS survey spectra were taken at an excitation energy of $\mathrm {h}\nu ={700}\,\hbox {eV}$ as illustrated in Fig. 3. An emission angle of $\Theta ={60}^{\circ }$ was chosen to achieve high surface sensitivity. For all spectra, including the clean Ag(111) sample at the bottom of Fig. 3, no residues could be identified in the sample systems, as typically represented by signals of O 1s and C 1s, whose expected energy locations are marked by the red boxes. The Ge film thicknesses of the respective phases is increased from bottom to top as displayed by the rising feature of the Ge 3d core-level at a binding energy of $E_\mathrm {bin} = {28.9}\hbox { eV}$.

**Figure 4:**
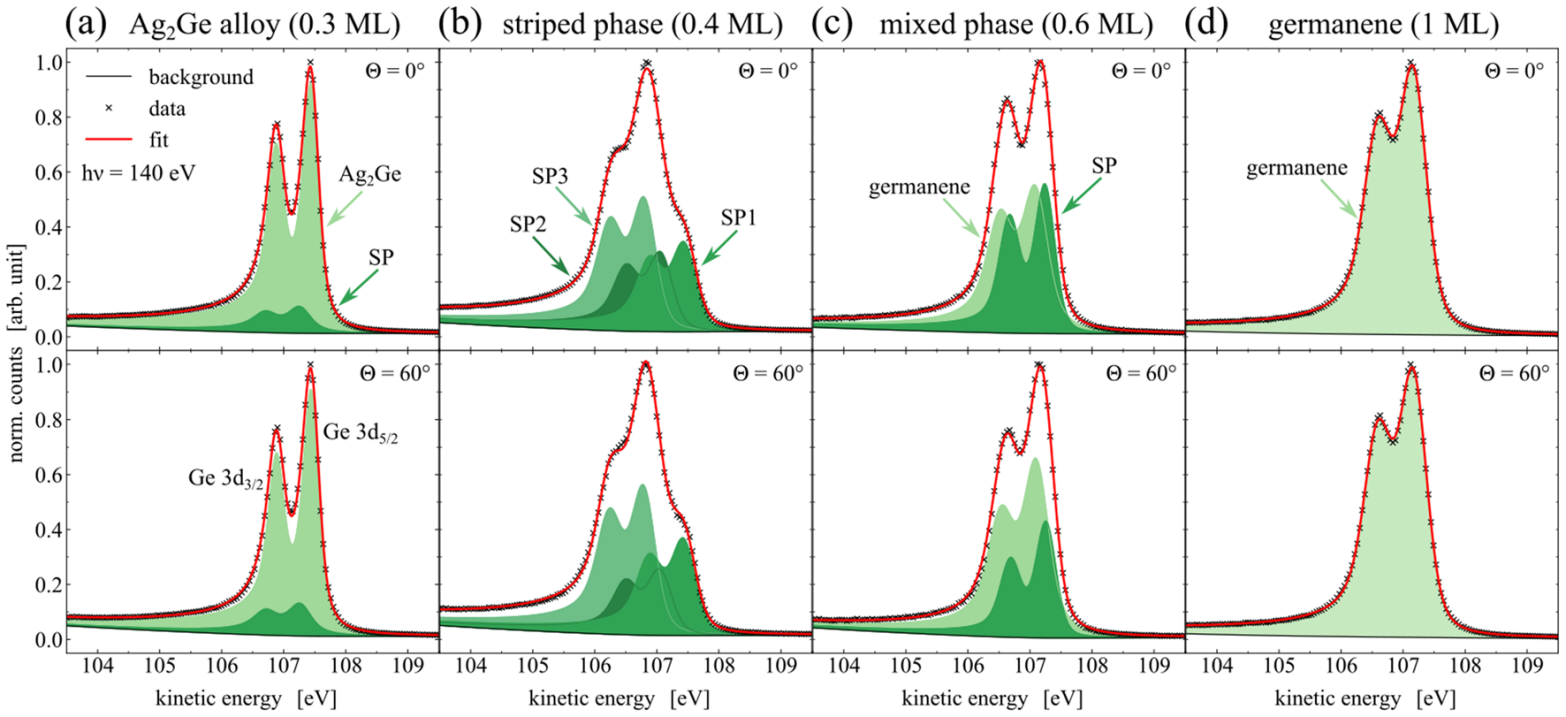
Analysis of the internal structure of all phases by means of high-resolution XPS spectra of the Ge 3d core-level. The spectra were taken at a photon energy of $\mathrm {h}\nu ={140}\hbox { eV}$ and under emission angles of $\Theta ={0}^{\circ }$ (top) and $\Theta ={60}^{\circ }$ (bottom). The fit parameters of each spectrum can be found in Table 2.

In order to resolve the internal and interfacial structure of the germanium phases, high-resolution XPS spectra of the Ge 3d and Ag 3d core-level were performed. The Ge 3d spectra are shown in Fig. 4, with each core-level measured under normal emission of $\Theta ={0}^{\circ }$ and at $\Theta ={60}^{\circ }$ with more sensitivity to the surface. The fit parameters yielded from the peak fitting as mentioned in “Methods” are listed in Table 2. The spectra which were obtained from the $\hbox {Ag}_2\hbox {Ge}$ alloy phase are displayed in Fig. 4a. The data were mainly fitted by one component with a spin-orbit separation of $E_\mathrm {SOC}={0.56}\,\hbox {eV}$. This component can be attributed to Ge atoms being embedded in the silver surface forming a $(\sqrt{3}\times \sqrt{3})$ surface alloy, as proposed by literature^25,26^. The low FWHM and a significant asymmetry indicates a high order and metal-like structure of the valence band^43^, respectively, which supports the model of embedded Ge atoms in a $\hbox {Ag}_2\hbox {Ge}$ surface alloy. The second SP component, which is shifted by $\Delta E_\mathrm {chem}={0.17}\,\hbox {eV}$ towards lower kinetic energies, represents a contribution of the striped phase^24,45^ and increases at slightly higher coverages. At an emission angle of $\Theta ={60}^{\circ }$ the intensity increases significantly by ${20}{\%}$, which indicates a near-surface arrangement compared to the atoms of the $\hbox {Ag}_2\hbox {Ge}$ component. Moreover, the broader FWHM of the SP component compared to the $\hbox {Ag}_2\hbox {Ge}$ component is in good agreement with the observed peak shapes of the striped phase fitting.

The internal structure of the striped phase corresponds to the XPS spectra in Fig. 4b. A notable change in the peak shape can be recognized by a composition of three upcoming components SP1, SP2, and SP3. The reported tensile strain of the uppermost layer in the striped phase causes the shortened bond lengths^32^ and modified chemical environments of the Ge atoms with respect to the surface. It leads to a larger unit cell of the superstructure than the unit cell of the $\hbox {Ag}_2\hbox {Ge}$ phase, as discussed in “LEED” on the basis of the obtained LEED-pattern in Fig. 2b. The proposed structure model by Zhang et al. allows to identify three groups of atoms^31^, each with different chemical environments, that can be attributed to the three components found in the XPS spectra. The asymmetry parameter of $\alpha >{0.1}$ for both alloy phases shows the metallic character of the Ge-formations, which might be a result of the strong interaction to the metallic Ag-substrate. A more detailed analysis of the interface structure between germanium and silver will be discussed later. With a maximum intensity increase of SP3 by ${8}{\%}$ and a decrease of SP2 by ${19}{\%}$ for high-emission angles $\Theta ={60}^{\circ }$, a buckling of the Ge atoms within the striped phases unit cell can be concluded.

The core-level signal of the Ge 3d orbital of the mixed phase is shown in Fig. 4c. As already indicated by the LEED pattern, its chemical structure consists of a mixture of the SP component representing the striped phase and a germanene component. The area ratio of both components is nearly 1 at normal emission, which indicates an almost equal amount of contributions from germanene and stripe phase inside the measuring field. A STM analysis of this phase performed by Lin et al. revealed the domain-like coexistence of both phases bounded by step edges, instead of a stacked layer growth of both phases^32^. However, the intensity of the germanene component increases by ${19}{\%}$ for high-emission angles with a simultaneously intensity decrease of the SP component by ${24}{\%}$. This observation is indicative for a topographic distribution of both phases. The germanene phase appears to be closer to the surface than the striped phase. While the Ge atoms of the striped phase are still embedded in the uppermost Ag layer, the germanene instead grows on top of the silver surface. STM observations show that the domains of the germanene phase are bounded from the stripe phase by step edges and grow mainly from the upper step edges^32^.

Figure 4d shows the core-level spectra of the germanene phase. The best fit was achieved by applying one single component to the data, which is supported by reports on a similar single-component peak shape of freestanding graphene^46,47^. The Ge atoms within the germanene honeycomb lattice are all located in the same chemical environment, which weakens a proposed highly buckled structure or a proposed coexisting $\hbox {Ag}_2\hbox {Ge}$ alloy phase with strong interaction to the Ag substrate^13,22^. A broadening of the FWHM can be noted, which corresponds to the *ordered disorder* of germanene on Ag(111), as also observed by STM^28,32,35^. A local disorder of the germanene is caused by domain rotations and compressive strain, while a long-range order is confirmed by the respective LEED-pattern^32,35^. However, as long as the asymmetry of XPS peaks is most likely for metallic materials^43^, the obtained asymmetry parameter of $\alpha ={0.08}$ for germanene can further indicate a rather $sp^2$-hybridized bonding structure in germanene^48,49^. Since the interaction with the substrate is weaker than in the case of the alloy phases, a metal-like behavior or $sp^2$-hybridized internal Ge-bonds commonly stand for a low-buckled formation^14^.

**Table 2:** Peak fit parameters of the Ge 3d signal resulting from the XPS analysis shown in Fig. 4.

Figure 4	$\Theta$ $(^{\circ })$	Comp.	$E_\mathrm {kin}$ $(\hbox {eV})$	$E_\mathrm {SOC}$ $(\hbox {eV})$	$\mathrm {FWHM}$ $(\hbox {eV})$	$\alpha$	$A_\mathrm {rel}$ $(\%)$
(a)	0	$\hbox {Ag}_2\hbox {Ge}$	107.45	0.56	0.33	0.12	86.7
		SP	107.28	0.56	0.33	0.12	13.3
	60	$\hbox {Ag}_2\hbox {Ge}$	107.46	0.56	0.50	0.12	84.0
		SP	107.29	0.56	0.50	0.12	16.0
(b)	0	SP1	107.46	0.56	0.50	0.13	29.7
		SP2	107.08	0.56	0.50	0.13	26.3
		SP3	106.82	0.56	0.50	0.13	44.0
	60	SP1	107.45	0.56	0.50	0.13	31.2
		SP2	107.07	0.56	0.50	0.13	21.2
		SP3	106.81	0.56	0.50	0.13	47.7
(c)	0	SP	107.26	0.57	0.40	0.08	43.7
		germ.	107.10	0.57	0.51	0.08	56.4
	60	SP	107.27	0.57	0.40	0.08	33.1
		germ.	107.12	0.57	0.51	0.08	66.9
(d)	0	germ.	107.18	0.57	0.54	0.08	100.0
	60	germ.	107.18	0.57	0.54	0.08	100.0

Each component consists of a convolution of a Doniach-Sunjic profile with asymmetry parameter $\alpha$ and a Gaussian.

**Figure 5:**
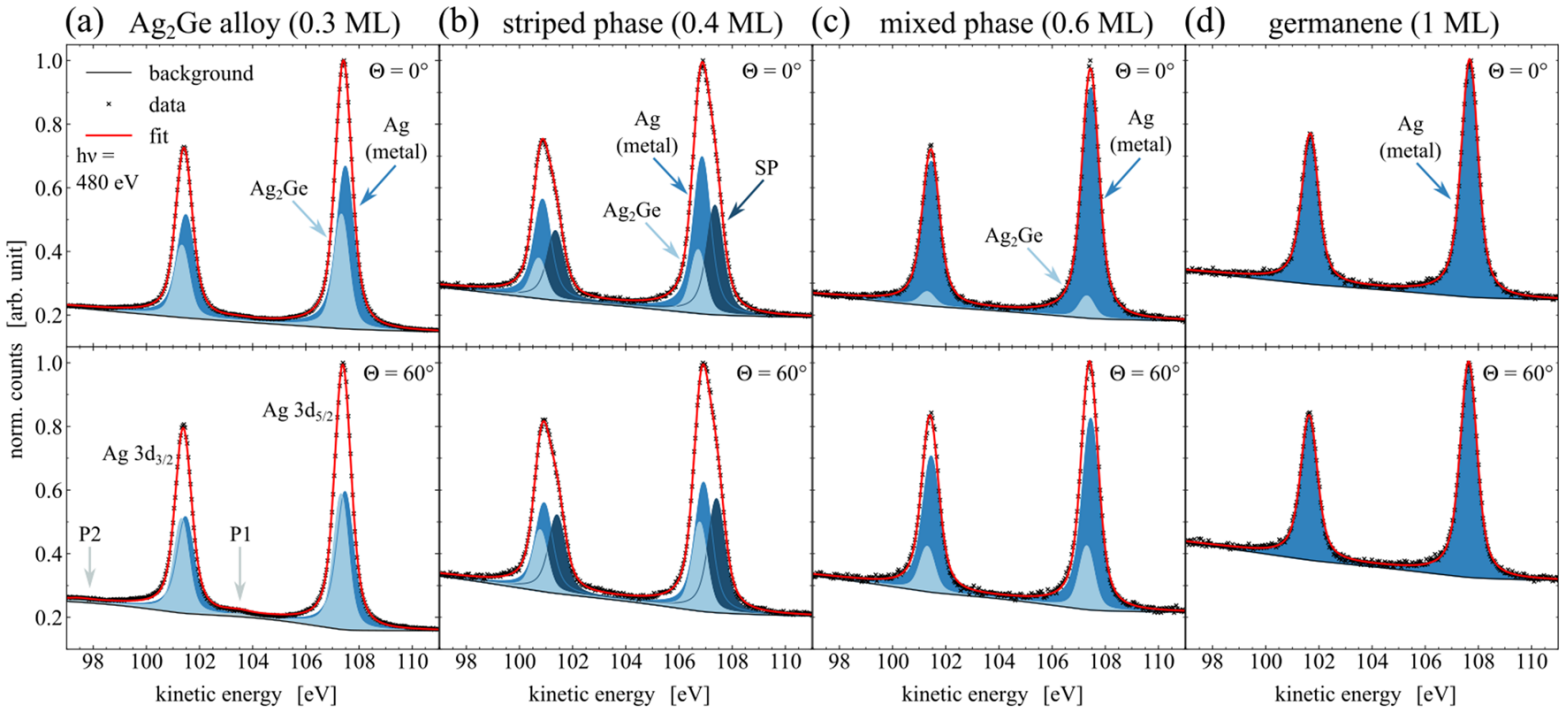
Analysis of the interfacial structure of all phases by means of high-resolution XPS spectra of the Ag 3d core-level. The spectra were taken at a photon energy of $\mathrm {h}\nu ={480}\hbox { eV}$ and under emission angles of $\Theta ={0}^{\circ }$ (top) and $\Theta ={60}^{\circ }$ (bottom). The fit parameters of each spectrum can be found in Table 3.

In order to examine the bonding structure at the Ge-Ag interface, high-resolution XPS measurements of the Ag 3d core-level were performed, as depicted in Fig. 5. Analogous to our measurements in Fig. 4, all spectra were recorded at emission angles of $\Theta ={0}^{\circ }$ and $\Theta ={60}^{\circ }$, here with an excitation energy of $\mathrm {h}\nu ={480}\hbox { eV}$. The corresponding fit parameters are provided in Table 3. Figure 5a displays the spectra of the Ag 3d signal obtained for the $\hbox {Ag}_2\hbox {Ge}$ surface alloy phase. The spectra reveal two components, chemically shifted by $\Delta E_\mathrm {chem}={0.15}\,\hbox {eV}$, which can be attributed to the metallic Ag, as comparable to Fig. 1a, and to an $\hbox {Ag}_2\hbox {Ge}$ alloy bond. These results support our proposed structure model of a high-ordered alloy phase, since even remaining features of surface plasmons could be identified. The $\hbox {Ag}_2\hbox {Ge}$ component represents a large share of the total intensity, which can be explained by the kinetic energy of the photoelectrons that results in a mean escape depth of $\lambda = {5.3}$ Å^50^. Furthermore, the intensities of the $\hbox {Ag}_2\hbox {Ge}$- and the metallic Ag-component change for recorded spectra under high emission by $+{21}{\%}$ and $-{30}{\%}$, respectively, which illustrates the $\hbox {Ag}_2\hbox {Ge}$ as surface component and the metallic Ag as bulk component.

In contrast to (a), the interface structure of the striped phase causes a remarkably different peak shape, as illustrated by the Ag 3d signal in Fig. 5b. In agreement with our observations in Fig. 4b, the upcoming SP component of the striped phase should result from the tensile strain of the top layer. The SP component, shifted by $\Delta E_\mathrm {chem}={0.48}\hbox { eV}$ towards higher kinetic energies with respect to the metallic Ag component, corresponds to Ag atoms with a reduced average binding length. In addition to the $\hbox {Ag}_2\hbox {Ge}$ component, both represent the silver atoms at the surface of the striped phase.

The depicted spectra in Fig. 5c were obtained for XPS core-level measurements of the mixed phase. Here, a peak shape is visualized, which is comparable to the $\hbox {Ag}_2\hbox {Ge}$ alloy phase in Fig. 5a, consisting of a $\hbox {Ag}_2\hbox {Ge}$- and a metallic Ag-component. In this case, the area ratio of the $\hbox {Ag}_2\hbox {Ge}$- to the Ag-component is reduced compared to the $\hbox {Ag}_2\hbox {Ge}$ phase. Since the mixed phase consists of contributions of the $\hbox {Ag}_2\hbox {Ge}$ phase and the germanene phase, it suggests that the silver internally forms metallic bonds at the surface, within the germanene phase.

Figure 5d displays the recorded XPS spectra of the Ag 3d signal for the germanene phase. As just mentioned, these spectra evince only a single component, which indicates a metallic state of the substrates atoms, as well as a low interaction at the interface between substrate and adsorbate. Besides the large background in both spectra due to the higher germanium coverage in comparison to the other phases, the peaks are shaped very similar to the peaks of the clean Ag(111) sample in Fig. 1a, supported by the obtained fit parameters in Table 3. In combination with our findings of the internal structure of germanene, as discussed for Fig. 4d, we conclude on a chemically freestanding germanene phase on Ag(111), since no strong interaction or structural deformation at the interface could be noted.

**Table 3:** Peak fit parameters of the Ag 3d signal resulting from the XPS analysis shown in Fig. 5.

Figure 5	$\Theta$ $(^{\circ })$	comp.	$E_\mathrm {kin}$ $(\hbox {eV})$	$E_\mathrm {SOC}$ $(\hbox {eV})$	$\mathrm {FWHM}$ $(\hbox {eV})$	$\alpha$	$A_\mathrm {rel}$ $(\%)$
(a)	0	Ag	107.49	6.00	0.78	0.02	63.1
		$\hbox {Ag}_2\hbox {Ge}$	107.34	6.00	0.67	0.02	36.4
		P1	103.44		1.10	0.00	0.4
		P2	97.44		1.11	0.00	0.0
	60	Ag	107.48	6.00	0.78	0.02	54.6
		$\hbox {Ag}_2\hbox {Ge}$	107.33	6.00	0.67	0.02	43.9
		P1	103.43		1.10	0.00	0.9
		P2	97.43		1.11	0.00	0.5
(b)	0	SP	107.36	6.00	0.76	0.02	33.7
		Ag	106.88	6.00	0.78	0.02	49.8
		$\hbox {Ag}_2\hbox {Ge}$	106.73	6.00	0.67	0.02	16.6
	60	SP	107.41	6.00	0.76	0.02	35.3
		Ag	106.93	6.00	0.78	0.02	41.3
		$\hbox {Ag}_2\hbox {Ge}$	106.78	6.00	0.67	0.02	23.4
(c)	0	Ag	107.46	6.00	0.78	0.02	92.7
		$\hbox {Ag}_2\hbox {Ge}$	107.31	6.00	0.67	0.02	7.3
	60	Ag	107.46	6.00	0.78	0.02	78.6
		$\hbox {Ag}_2\hbox {Ge}$	107.31	6.00	0.67	0.02	21.4
(d)	0	Ag	107.68	6.00	0.77	0.02	100.0
	60	Ag	107.64	6.00	0.77	0.02	100.0

Each component consists of a convolution profile of a Doniach-Sunjic distribution with asymmetry parameter $\alpha$ and a Gaussian.

### XPD

**Figure 6:**
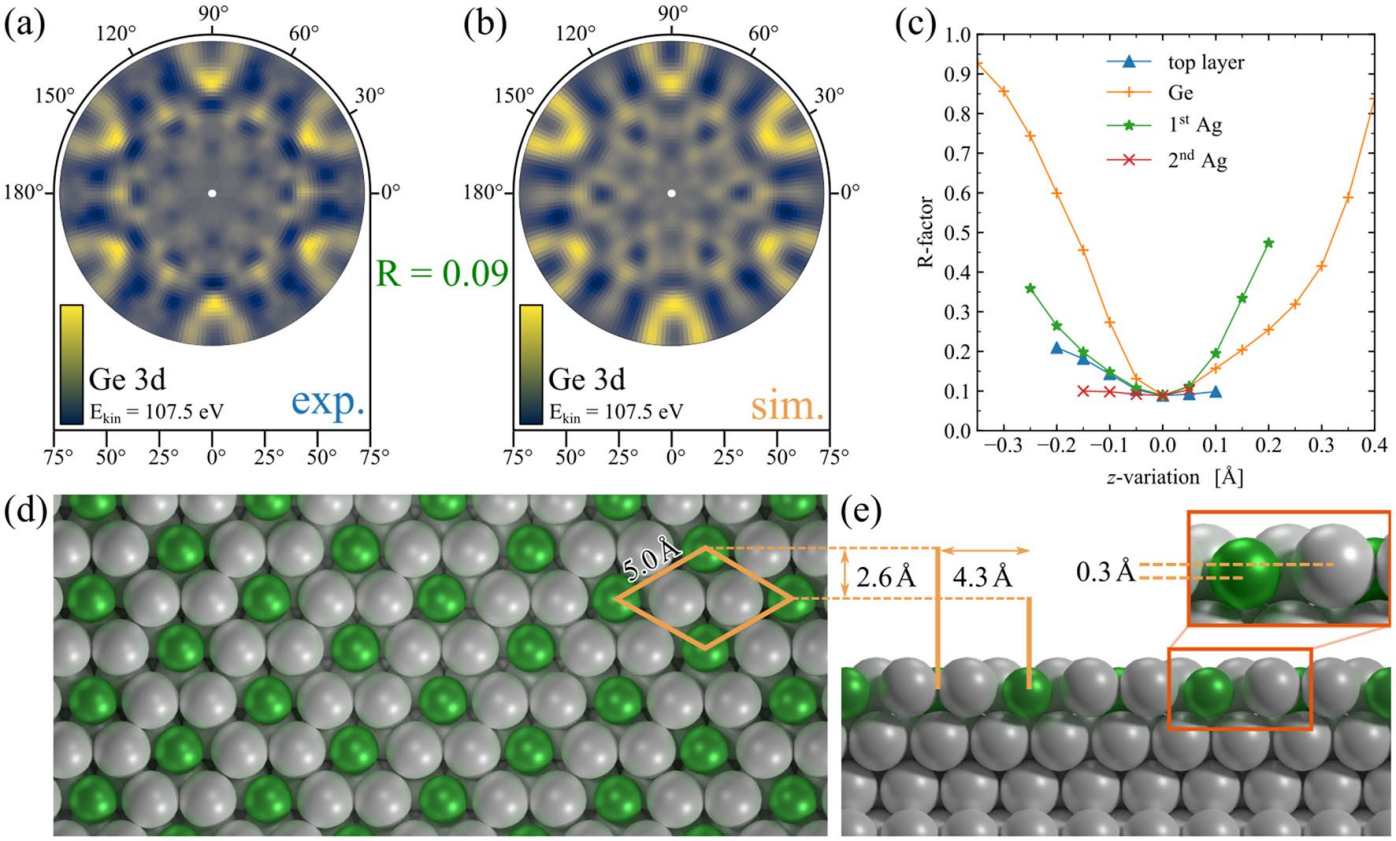
XPD analysis of the $\hbox {Ag}_2\hbox {Ge}$ alloy phase. (**a**) Experimental and (**b**) simulated XPD-pattern for the Ge 3d emitter of the $\hbox {Ag}_2\hbox {Ge}$ surface alloy phase, with a kinetic energy of $E_\mathrm {kin}={107.5}\,\hbox {eV}$. The minimum of the R-factor of $\mathrm {R} = 0.09$ was checked in (**c**). The corresponding best fitting structure is depicted in (**d**) top view and (**e**) side view. The green and silver spheres represent the Ge and Ag atoms, respectively.

In order to determine the specific structural assembly of the initial $\hbox {Ag}_2\hbox {Ge}$ surface alloy and the final germanene phase, we performed XPD measurements and simulations of the Ge 3d core-level. For all measured patterns a background subtraction, a three-fold rotational symmetry, a mirror symmetry, and a Gaussian blur were applied. Figure 6 represents the XPD analysis of the $\hbox {Ag}_2\hbox {Ge}$ phase, while Fig. 6a,b show the original experimental pattern and the subsequent simulated XPD pattern, respectively. A structural model for the $(\sqrt{3}\times \sqrt{3})\mathrm {R}30^\circ$ reconstruction of the $\hbox {Ag}_2\hbox {Ge}$ phase was developed based on our LEED and XPS analysis, as well as supported by proposed structural models by Golias et al. and Liu et al.^25,26^. With the help of a genetic algorithm, we found the best fitting test structure, which is illustrated in Fig. 6d,e. The corresponding simulated pattern agrees with the experimentally obtained pattern as indicated by the very low R-factor of $\mathrm {R} = 0.09$.

The stability of the R-factor minimum was tested by applying small variations in translation of some cluster groups to the final structure. In this case, the *z*-location of the topmost surface layer, the Ge atoms, and the first and second layer exclusively of Ag atoms were varied in order to carry out simulations for each test structure. Their R-factors were plotted in Fig. 6c which confirm the minimum R-factor for this formation. Moreover, the yielded structural parameters of a lattice constant of 5.0 Å and an embedding depth of 0.3 Å are in good agreement with published observations^26,28^.

**Figure 7:**
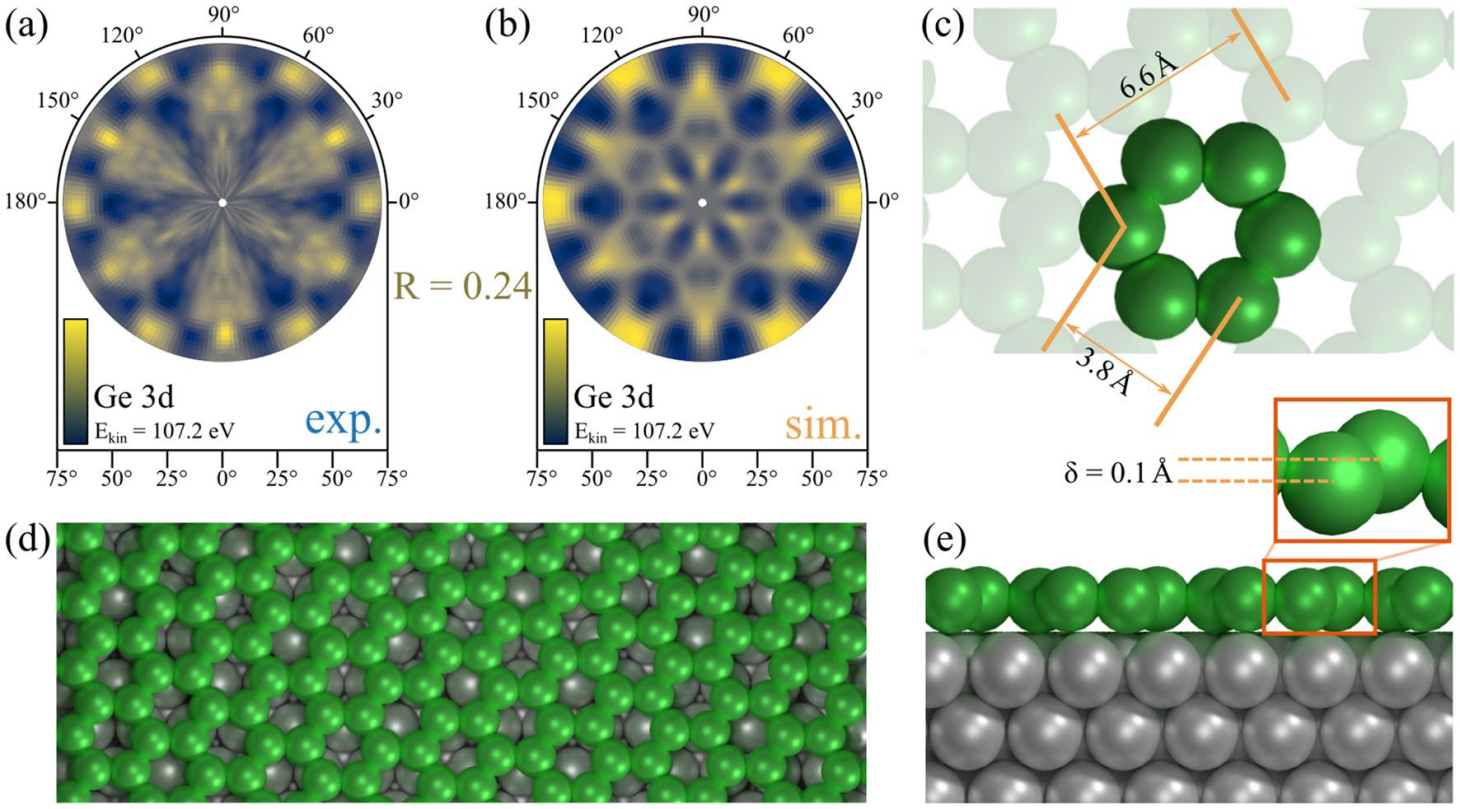
XPD analysis of the germanene phase. (**a**) Experimental and (**b**) simulated XPD-pattern for the Ge 3d emitter of the quasi-freestanding germanene phase, with a kinetic energy of $E_\mathrm {kin}={107.2}\,\hbox {eV}$. An R-factor analysis was carried out in Fig. 8, taking various structural models into account. The best fitting structure, shown in (**c**–**e**), returns the simulated pattern in (**b**) with an R-factor of $\mathrm {R} = 0.24$.

**Figure 8:**
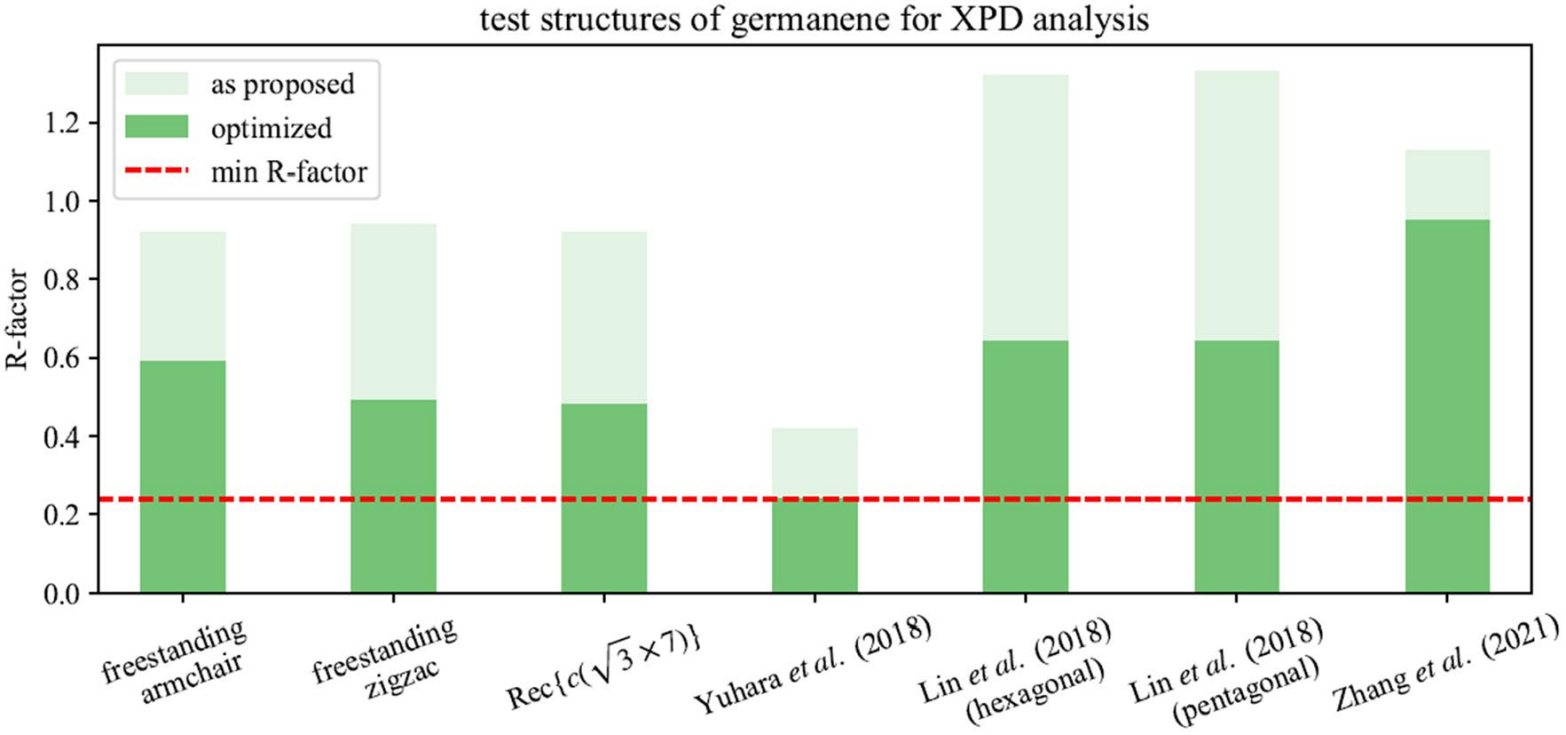
Tested structures of germanene for XPD analysis. Overview of XPD simulations for proposed test structures of germanene on Ag(111), before and after modification by the genetic algorithm. The tested structure models are based on theoretical structures for freestanding germanene and recent publications^10,31–34^.

Furthermore, an XPD analysis of the germanene phase was performed, as shown in Fig. 7. Subfigure (a) displays the experimental pattern. The variation of the anisotropy function $\chi$ of the diffraction pattern is much lower in this case, compared to the alloys pattern. As already mentioned, an *ordered disorder* of the germanene phase can be identified in real space images^27,28,32^, that both causes broad components in XPS spectra^35^ and the here observed blur of the diffraction maxima in XPD pattern, as well as a poor anisotropy range. Additionally, the periodicity of the germanene superstructure is expected to be ${5.35}\hbox { nm}$, as proposed by Yuhara et al.^34^, which results in a huge unit cell, that is close to the experimental limit of the XPD method. Nevertheless, several structures were tested for simulations to agree with the experimental XPD pattern. Figure 8 gives an overview of the resulting R-factors after the simulation of each structure as proposed and after the optimizing modification by the genetic algorithm. The lowest R-Factor of $\mathrm {R} = 0.24$ was obtained for a modified structure starting from the proposed structural model by Yuhara et al.^34^. Figure 7b depicts the simulated pattern, showing similar features of maxima and minima at the same directions $(\phi , \Theta )$. The corresponding structure is illustrated in Fig. 7d,e, while (c) displays a close-up of a single hexagon within the honeycomb lattice. The germanene superstructure satisfies a $(7\sqrt{7}\times 7\sqrt{7})\mathrm {R}19.1^\circ$ with respect to the Ag(111) surface^34^. The lattice constant of freestanding germanene was calculated to be 3.97 Å with a buckling distance of $\delta = {0.64}$ Å^10^. However, Yuhara et al. proposed a lattice constant of 3.9 Å with a buckling of $\delta = {0.1}$ Å from their investigations which results in a unit cell size of ${5.35}\hbox { nm}$^34^.

Our XPD analysis returned structural parameters as a lattice constant of 3.8 Å, a period length of 6.6 Å, as well as a low buckling of $\delta = {0.1}$ Å. The minimum spacing between adsorbate and substrate amounts 2.6 Å, which supports our observations from XPS regarding the weak interface interaction. All these parameters are in excellent agreement with further investigations of germanene on Ag(111)^32,34^ and on other substrates^20,51^. Based on our investigation, we determine the germanene phase on Ag(111) to be *quasi*-freestanding, because its structural parameters differ from theoretical models of freestanding germanene. However, we recovered a surprisingly low buckled honeycomb structure, that is very well comparable to a germanene phase which was grown via segregation through Ag(111)^34^.

## Conclusion

We presented the structural evolution of epitaxially grown germanene on Ag(111). Four different formations were determined with respect to their superstructure as observed by LEED measurements. A first $\hbox {Ag}_2\hbox {Ge}$ alloy phase at a coverage of $1/3\,\hbox {ML}$ forms a $(\sqrt{3}\times \sqrt{3})\mathrm {R}30^\circ$ reconstruction before turning into a more complex superstructure of $\mathrm {Rec}(\sqrt{3}\times 17)$ due to a tensile strain of the uppermost layer of the surface alloy. The mixed phase is a transition phase which shows a superposition of the particular LEED pattern of the coexisting surface alloy and the $\mathrm {Rec}\{c(\sqrt{3}\times 7)\}$ reconstruction of the germanene formation. Additionally, high-resolution core-level spectroscopy of the Ge 3d and the Ag 3d orbital was performed, revealing a clear distinction between all phases. In particular, the germanene phase was observed to be chemically decoupled from the substrate resulting in a freestanding layer. XPD measurements and according simulations of test structures led to precise structure models of the $\hbox {Ag}_2\hbox {Ge}$ surface alloy, as well as the freestanding germanene. Here, quasi-freestanding germanene was revealed with a ${4}{\%}$ smaller lattice constant as proposed by theory, as well as a low buckling of $\delta = {0.1}$ Å.

The detailed interface analysis within our study cleared up the controversial discussion about the alloy phases at a coverage of $1/3\,\hbox {ML}$ by our proposed internal and interfacial structure for the $\hbox {Ag}_2\hbox {Ge}$ phase and the striped phase. Additionally, we proofed Ag(111) to be a promising substrate for the growth of freestanding germanene. However, our structural determination revealed a rather metal-like germanene by its flat honeycomb formation and the $sp^2$-hybridized Ge bonds. This work progresses the fundamental understanding of the structural formation of 2D-materials, using the example of germanene on Ag(111).

## Methods

All sample preparation stages and measurements were performed in-situ in an ultra-high vacuum (UHV) chamber with a base pressure of $p=5\times 10^{-11}\hbox {mbar}$. The chamber provides a sputter gun, a heating stage, a 4-grid LEED system, and a hemispherical energy analyzer for the detection of photoelectrons. The 5-axis manipulator allows to move the sample in *x*-, *y*-, and *z*-direction, as well as continuous rotations of the azimuthal axis $\phi$ and wide-range rotations of the polar axis $\Theta$ with respect to the surface normal. This UHV-chamber represents the endstation of beamline 11 at the DELTA electron storage ring of TU Dortmund University. The beamline provides linearly polarized soft X-ray radiation from the undulator U55 in an energy range of ${55}\,\hbox {eV}\le \mathrm {h}\nu \le {1500}\,\hbox {eV}$, which is freely tunable by a plane-grating monochromator^52^.

The preparation of a pristine and reconstructed Ag(111)-surface was achieved by several cycles of Argon-ion bombardment with $E_\mathrm {kin}={600}\hbox { eV}$ and annealing at ${720}\hbox {K}$. The cleanness and long-range order of the sample was checked by XPS survey spectra and LEED measurements. Germanium phases of different layer thicknesses were grown epitaxially on the clean Ag(111)-surface by means of physical vapor deposition (PVD), using an electron beam evaporator. The linear evaporation rate was estimated from quartz crystal microbalance measurements as approximately 7 Å$\hbox { h}^{-1}$. During the evaporation, the Ag(111)-sample was kept at a temperature of ${420}\hbox { K}$, while continuously rotating around the $\phi$-axis to obtain a homogeneous growth of the layers. All found phases were investigated by LEED and XPS. The XPS spectra with high energy-resolution of the Ge 3d and Ag 3d core-levels were recorded at photon energies of $\mathrm {h}\nu = {140}\,\hbox {eV}$ and $\mathrm {h}\nu = {480}\,\hbox {eV}$, respectively. Each core-level was analyzed under normal emission angle of $\Theta ={0}^{\circ }$ and high emission angle $\Theta ={60}^{\circ }$. In a first step, the obtained XPS data were normalized to their maximum. Then, a removal of the background was conducted in the shape of a Tougaard background for the Ag 3d and the Ge 3d signal^53^. The peak shape is modelled by a convolution profile of a Doniach-Sunjic distribution with an asymmetry parameter $\alpha$ and a Gaussian function^54^. The profiles are applied to the data using a fit routine as implemented in the program *UNIFIT 2022*^55^. The given tables for the respective XPS figures provide each resulting fit parameters referring to the 3d$_{5/2}$ peak, since parameters like the full width at half maximum (FWHM) and the asymmetry parameter are in this case identical for both branches 3d$_{3/2}$ and 3d$_{5/2}$. Further, XPD measurements were performed for the $\hbox {Ag}_2\hbox {Ge}$ alloy phase and germanene phase. The core-level photoelectrons propagate through the solid with spherical electron wave character, while they are scattered elastically at neighboring atoms. The interference pattern caused by single and multiple scattering events can be observed as a modulation of the signals’ intensities depending on the emission angle^56,57^. For this purpose, diffraction pattern of the Ge 3d core-level were recorded over a polar angle range of ${2}^{\circ }\le \Theta \le {72}^{\circ }$ with an angular increment of $\Delta \Theta ={2}^{\circ }$ and an azimuthal angle range of ${0}^{\circ }\le \phi <{360}^{\circ }$ with a step width of $\Delta \phi ={1.8}^{\circ }$. In total, the presented XPD patterns consist of 7200 single XPS spectra, whose integrated intensity corresponds to the colorscaled pixels. Additionally, a normalizing anisotropy function per polar angle $\Theta$ is applied to the pattern,

1 $$\begin{aligned} \chi \left( \Theta ,\phi \right) = \frac{I(\Theta ,\phi )-\overline{I(\Theta )}}{\overline{I(\Theta )}}\;, \end{aligned}$$

where $I(\Theta ,\phi )$ is the measured intensity at $(\Theta ,\phi )$ and $\overline{I(\Theta )}$ is the average intensity for each polar angle $\Theta$. To determine the structure of the investigated sample, XPD simulations of test structures are carried out using the *EDAC* simulation package^58^. The comparison between experimental and calculated pattern quantifies the agreement of the samples structure and the test structure^59,60^. An adequate quality criterion for the agreement check is represented by the reliability factor (R-factor) defined as^61^,

2 $$\begin{aligned} \mathrm {R} = \frac{\sum _{\Theta ,\phi }\left[ \chi _\mathrm {exp}(\Theta ,\phi )-\chi _\mathrm {sim}(\Theta ,\phi )\right] ^2}{\sum _{\Theta ,\phi }\left[ {\chi _\mathrm {exp}}^2(\Theta ,\phi )+{\chi _\mathrm {sim}}^2(\Theta ,\phi )\right] }\;. \end{aligned}$$

The R-factor analysis yields values in the range of $0\le \mathrm {R}\le 2$, while $\mathrm {R}=0$ indicates a perfect alignment, $\mathrm {R}=1$ means an independency of the patterns, and $\mathrm {R}=2$ is obtained for totally anti-correlated patterns. To minimize the R-factor within a structural analysis process, the simulation is attached to a genetic algorithm, which applies variations and mutations to the initial test structure in order to minimize the R-factor and to approach the samples structure^62,63^.

## Data Availability

The data that support the findings of this study are available upon reasonable request from the authors.
